# Foreign Body Appendicitis Coexisting With Ascariasis in a Pediatric Patient: A Case Report

**DOI:** 10.7759/cureus.59632

**Published:** 2024-05-04

**Authors:** Ghazanfar Khan, Shakeel Khan, Muhammad Idrees, Hamza Khan, Hajra Faheem

**Affiliations:** 1 Department of General Surgery, Mardan Medical Complex, Mardan, PAK; 2 Department of Radiology, Mardan Medical Complex, Mardan, PAK; 3 Department of Pediatrics, Mardan Medical Complex, Mardan, PAK

**Keywords:** case report, foreign body ingestion, pediatric patient, ascariasis, foreign body appendicitis

## Abstract

In clinical practice, the typical approach to ingested foreign bodies in stable patients involves expectant management, as most materials pass through the gastrointestinal (GI) tract without adverse effects. However, foreign bodies that travel through the appendix's lumen can cause acute appendicitis due to their inability to exit the colon. Rarer causes of appendicitis include parasitic infiltration by *Ascaris lumbricoides*. The wandering behavior of *Ascaris lumbricoides* within the GI tract can lead to various surgical complications in the abdomen. Occasionally, these parasites can migrate to the vermiform appendix, where they may either induce pathological changes or remain asymptomatic.

We report an unusual case of an eight-year-old Pakistani female patient who presented to the emergency room with pain in the right iliac fossa, associated with anorexia and nausea, for one day. On examination, the patient was found to be vitally stable, with right iliac fossa tenderness noted on palpation. Additionally, the patient exhibited positive pointing, rebound, Rovsing, and psoas signs. Her medical history revealed that she had ingested a metallic needle seven months ago. Blood tests were undertaken, and an abdominal X-ray confirmed the existence of a radiopaque metallic object in the right lower quadrant of the abdomen. The patient underwent an open appendicectomy for acute appendicitis and was discovered to have a metallic needle lodged in the vermiform appendix. Concurrently, she also had ascariasis, as she vomited a 23-cm-long *Ascaris lumbricoides* worm.

It is important to consider both mechanical and parasitic etiologies in diagnosing acute appendicitis; detailed evaluation and management strategies are necessary to address these unique etiologies effectively.

## Introduction

The appendix, often considered a vestigial organ, is typically located at the tip of the cecum in the right lower quadrant of the abdomen and is prone to inflammation, a condition known as acute appendicitis [1]. Appendicitis is not exclusive to any specific age group but is most prevalent among individuals aged 10 to 20 years. Foreign body-induced appendicitis is commonly found in pediatric patients. Statistically, males are more susceptible to this condition than females, with a lifetime risk of 8.6% for males and 6.9% for females [2]. The cause of acute appendicitis is unidentified in about 60% of cases, while in the remaining instances, it is typically due to lumen obstruction, commonly attributed to fecal matter or lymphoid hyperplasia; more infrequently, tumors, intestinal parasites, or foreign bodies are responsible. The occurrence of acute appendicitis associated with foreign bodies is very rare, with a prevalence rate of 0.0005% [3].

In the literature, various foreign objects have been documented as causing acute appendicitis. These include inedible and non-digestible items commonly found in children, such as balls, fishing lines, screws, coins, stones, toothbrush hair, pins, needles, teeth, dog hair, toothpicks, strands, tongue studs, a crown rod, lead shot, a die, the end of a thermometer, a pen tip, stones, and keys. Adults have experienced appendicitis due to intrauterine devices, condoms, and metal objects like razor blades. Additionally, edible and digestible foreign bodies like fruits, seeds, fish bones, and bone fragments have also been implicated [4].

The prevalence of appendicitis linked to helminthic infection varies widely, reported between 1.5% and 27.2%, with a notably higher incidence in pediatric populations. The involvement of *Ascaris lumbricoides*, a common intestinal parasite, in causing appendicitis is controversial; while it can migrate to the appendix, mimicking appendicitis symptoms, its role as a direct causative agent remains unclear [5]. Suspected appendicitis due to roundworm is commonly confirmed through the identification of roundworm eggs in the appendectomy specimen [6]. Wani et al. found in their study that while 72.7% of patients with *Ascaris lumbricoides* underwent surgical treatment, only 27.2% showed histopathological evidence of appendicitis linked to *Ascaris lumbricoides* [7].

In line with Surgical CAse REport (SCARE) guidelines, we report a case of foreign body appendicitis, discovered intraoperatively, coexisting with ascariasis in an eight-year-old child who vomited a 23-cm-long *Ascaris lumbricoides* worm [8]. This case report highlights the rare occurrence of foreign body appendicitis alongside ascariasis in a pediatric patient, offering valuable insights into the diagnostic complexities and treatment approaches of this dual condition.

## Case presentation

An eight-year-old Asian-Pakistani female patient, with a body mass index (BMI) of 14.5 kg/m^2^, belonging to a family with a satisfactory economic status, presented to us in the emergency department with chief complaints of pain in the abdomen, associated with anorexia and nausea for one day. The pain was colicky in nature and started around the umbilicus initially and then shifted to the right iliac fossa.

Upon taking a history, it was revealed that the patient had ingested a metallic needle seven months ago. Following the ingestion, the parents decided to monitor the condition as recommended by the healthcare provider without opting for surgical intervention or medication. The patient had a negative surgical history, no relevant drug history, and no known allergies to drugs or food. There was no significant family history of major diseases.

On examination, the patient was vitally stable, and no pallor or jaundice was noted. On palpation, the abdomen was tender in the right iliac fossa, and the patient had positive pointing, rebound, Rovsing, and psoas signs. During bedside assessment, blood pressure was 117/78 mmHg, pulse rate was 86 beats per minute (bpm), oxygen saturation was 98% on room air, and she was afebrile.

Laboratory investigations, including a complete blood count and serum electrolytes, were carried out, as detailed in Table 1.

**Table 1:** Laboratory investigations performed pre- and postoperatively WBC: white blood cells; Neu: neutrophils; Eos: eosinophils; Hb: hemoglobin; PLT: platelets

Investigation	Reference value	Results (preoperative)	Day 1 (postoperative)
WBC	4-10 10^^3^/ul	12.2	10.5
Neu	2-7 10^^3^/ul	10	9.7
Eos	0.02-0.50 10^^3^/ul	0.29	0.25
Hb	11.0-16.0 g/dl	12.9	12.7
PLT	150-450 10^^3^/ul	436	442
Sodium (Na)	135-148 mmol/L	139	140
Potassium (K)	3.6-5.2 mmol/L	4.03	4.1
Chloride (Cl)	98-108 mmol/L	103	105

**Figure 1:**
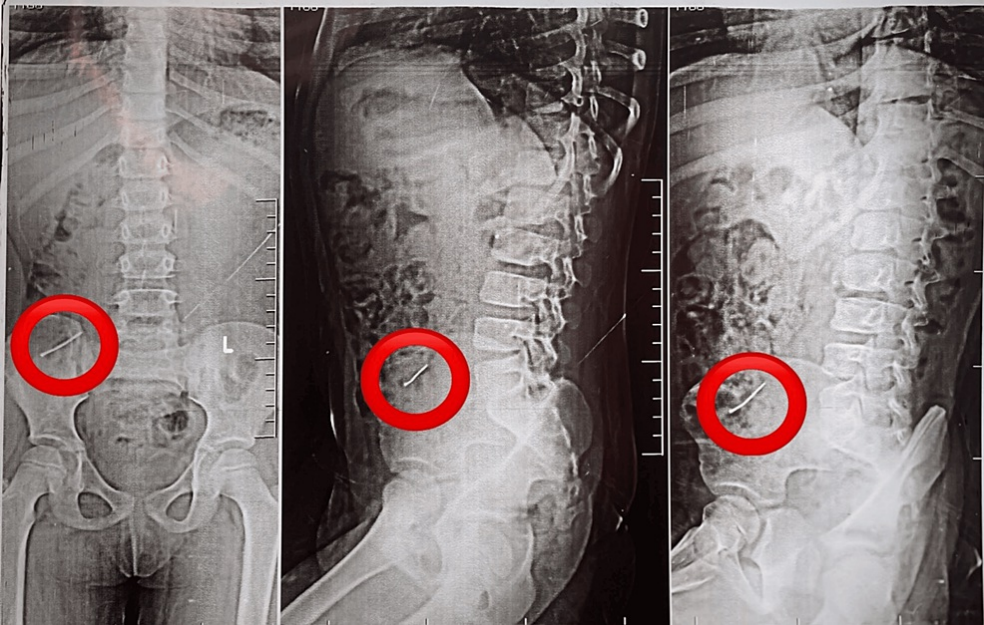
A plain film X-ray shows a radiopaque foreign body in the right iliac fossa

The viral profile, including hepatitis B surface antigen (HBsAg), human immunodeficiency virus (HIV), and hepatitis C virus (HCV), was negative, and the urine routine examination was normal. The imaging study included abdominal X-rays, which revealed a sharp radiopaque foreign body located in the right iliac fossa (Figure 1).

**Figure 2:**
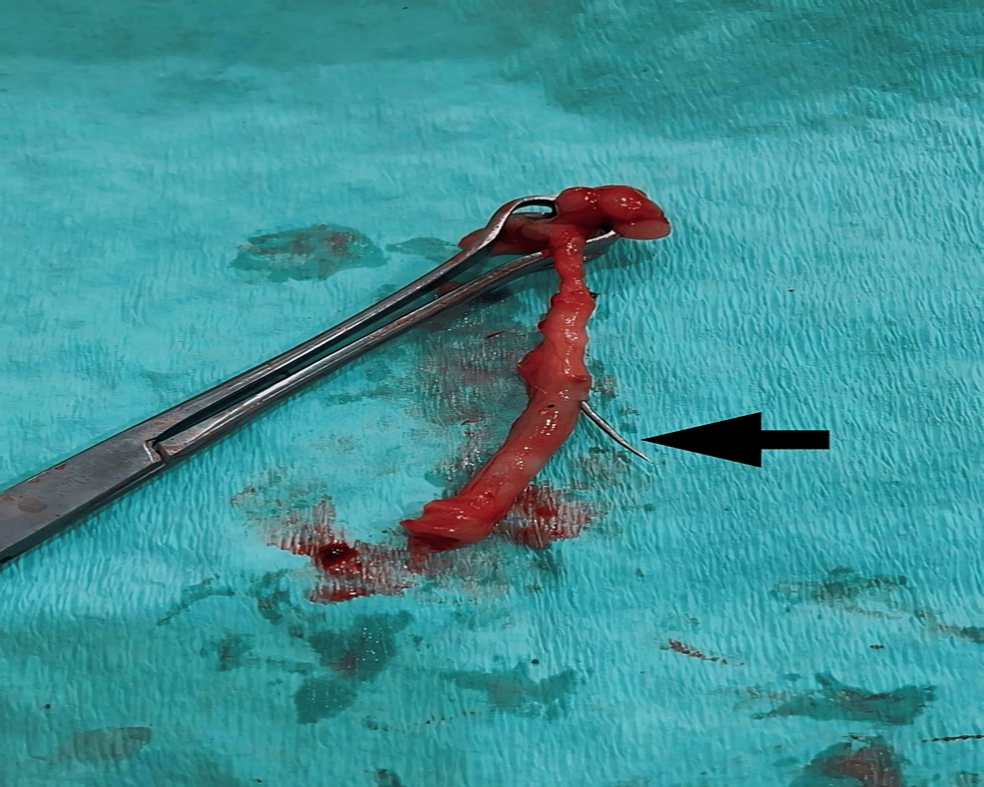
Gross view of the appendix post appendicectomy with the needle penetrating the appendix

The parents of the patient were counseled regarding the course of the disease and a nil-by-mouth (NBM) status was maintained for six hours preoperatively. The patient received an infusion of paracetamol 30 ml intravenously (IV) stat for pain relief and injection of ceftriaxone 500mg IV stat for antibiotic prophylaxis preoperatively. Following completion of the preoperative checklist, the patient was transferred to the operating room. Under the aseptic technique, the patient was appropriately draped, and general anesthesia was administered by a skilled anesthesiology team. After sedation, a Gridiron skin incision was given, and the abdominal cavity was opened layer by layer. The appendix was identified followed by ligation of the mesoappendix and base of the appendix with Vicryl 1 suture and cut separately. The appendix was retrieved and on inspection, it was discovered that a sharp metallic needle was present in the appendix lumen (Figure 2). The hemostasis was secured, and the abdomen was closed in a reverse fashion with Vicryl 1 suture. The skin was closed in vertical mattress fashion with Prolene 2/0 suture, and an aseptic dressing was done. The patient’s recovery from general anesthesia was uncomplicated and she was mobilized and allowed a liquid diet six hours after surgery.

**Figure 3:**
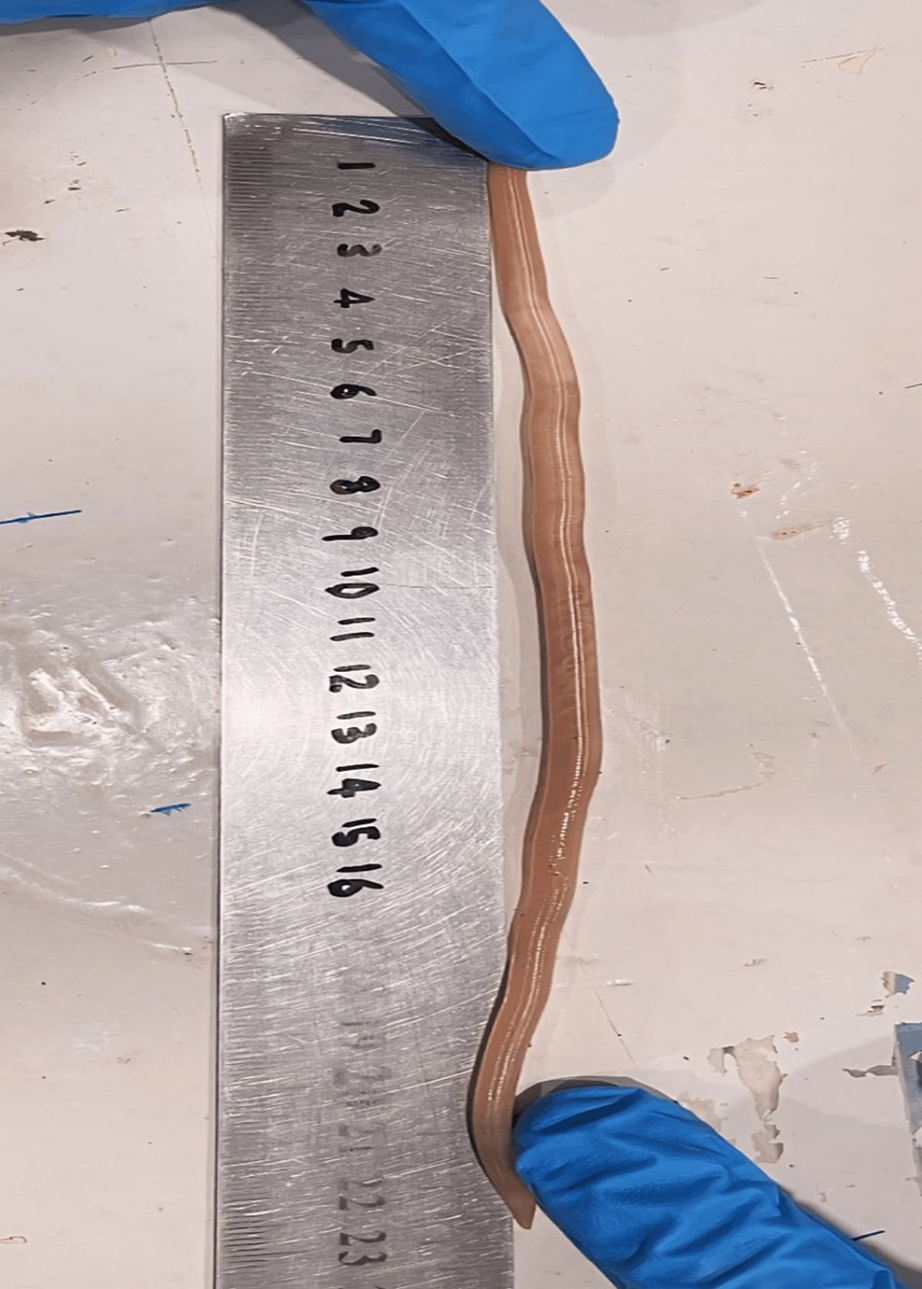
Gross view of the Ascaris lumbricoides worm vomited by the patient

On the first postoperative day, the patient vomited an approximately 23 cm long *Ascaris lumbricoides worm* (Figure 3). The parents denied any prior occurrence of similar events. An abdominal ultrasonography was performed, which did not show any signs of parasitic infestation.

The patient was observed in the surgical unit for 48 hours postoperatively, and the hospital medications included an infusion of paracetamol (30 mL) IV thrice daily, an injection of ceftriaxone (500 mL) IV twice daily, an infusion of metronidazole (30 mL) IV thrice daily, and an infusion of Plabolyte-M (500 mL) IV twice daily. The patient was discharged home with oral medication, including a syrup of paracetamol 250mg one teaspoon thrice daily for five days, a syrup of co-amoxiclav 457mg/5ml one teaspoon twice daily for five days, and a syrup of albendazole 200 mg single dose, along with a dietary instruction to consume fruits and vegetables and avoid spicy foods. A two-week follow-up plan was advised to the patient in the surgical outpatient department.

The patient diligently followed the advised diet and medication regimen. The postoperative recovery was excellent, with no reported intraoperative or postoperative complications.

## Discussion

Appendicitis is a common surgical emergency with a global incidence rate of 100 cases per 100,000 individuals across all age groups, making it one of the most prevalent acute abdominal conditions [9]. Acute appendicitis due to foreign bodies is a rare phenomenon, with an extremely low prevalence rate of only 0.0005% [1]. In a study involving 71,000 cases of appendectomies, it was found that 51.8% of the cases were linked to obstruction, primarily caused by parasitic worms or faecoliths, while only 5.5% were associated with unusual foreign bodies [3].

Pathophysiologically, when the weight of a foreign body surpasses the content of the bowel fluid, it becomes lodged in the lower portion of the cecum, where it accumulates. Once it enters the lumen of the appendix, the peristaltic movements are inadequate to push the foreign object back into the cecum [1]. Due to the dependent position of the cecum, foreign bodies tend to settle down, particularly heavier objects. Subsequently, the appendiceal orifice may widen to accommodate the lodging of these foreign bodies into its lumen. However, this process is unlikely to occur in cases involving a retrocecal appendix [10].

In foreign body appendicitis, the complications can vary depending on the size and shape of the foreign body. Blunt objects tend to block the appendiceal lumen, leading to appendicitis, while sharp objects are more prone to causing perforation along with appendicitis [10].

Roundworms, specifically *Ascaris lumbricoides*, derive their name from their resemblance to earthworms. These parasites are typically transmitted through the fecal-oral route, primarily by ingesting water or food, particularly vegetables and fruits, that are contaminated with their eggs [11]. *Ascaris lumbricoides*, being a highly mobile parasite, can invade the biliary and pancreatic ducts, potentially causing conditions such as biliary colic, cholangitis, acalculous cholecystitis, and acute pancreatitis. While it can migrate to the appendix, mimicking appendicitis symptoms, its role as a direct causative agent remains unclear. However, its migration to the stomach is rare, likely attributed to the harshly acidic environment and robust peristaltic movements within the stomach [5, 12].

When managing foreign body ingestion, the initial steps involve imaging and a prompt upper endoscopy to locate and extract the foreign object if possible. If endoscopic retrieval is unsuccessful, follow-up with a plain abdominal X-ray is necessary to monitor the foreign body's progression in the gastrointestinal (GI) tract. Radiographic signs to look for include gas in the appendix, distension of the terminal ileum or cecum, and an ascending colon. Additional imaging modalities, such as ultrasound, may be used to confirm foreign body-related appendicitis, potentially reducing unnecessary hospitalizations and surgeries. A complete blood count can help detect leukocytosis, particularly in asymptomatic cases. Although ultrasound is valuable in assessing appendicitis, its effectiveness in diagnosing helminth infestation is limited [5, 13].

The primary treatment goal is to reduce the risk of complications, especially perforation, which is more prevalent in cases involving sharp and elongated foreign objects, often requiring appendectomy. In some situations, monitoring the natural passage of foreign bodies can be an alternative strategy [1]. In preoperative diagnoses, surgical removal of the appendix, using either an open or laparoscopic technique, is the standard management approach without specific preoperative considerations [14]. Antiparasitic therapy, typically with medications like albendazole or mebendazole, should commence upon confirming parasitic infestation. Additionally, patient and family education on hygiene and sanitation practices is crucial to preventing future infections [5].

Our case is significant for understanding appendicitis and its rare causes, including foreign bodies and parasites. Acute appendicitis is a prevalent surgical emergency globally, underscoring the importance of prompt diagnosis and treatment to prevent potential complications.

## Conclusions

Our case highlights the importance of considering parasites and asymptomatic foreign body ingestion as causes of appendicitis, despite the temporal gap between foreign body ingestion and appendicitis. Adequate prophylactic measures and precise imaging techniques are vital based on the ingested object and the patient's health status. It is crucial to thoroughly evaluate children with abdominal signs and symptoms to rule out any parasites or foreign objects that could be causing appendicitis. Early recognition and proper management play a critical role in preventing complications and optimizing patient outcomes. Further research and clinical awareness are essential for advancing the understanding and management of these distinct manifestations of appendicitis.

## References

[1] Hamadneh M, Al-Khalaileh M, Alayed A, Barhoush FR, Hijazin S, Haddad J, Abu-Jeyyab M (2023). Previous foreign body ingestion in the appendix causing acute appendicitis: a case report. Cureus.

[2] Baird DL, Simillis C, Kontovounisios C, Rasheed S, Tekkis PP (2017). Acute appendicitis. BMJ.

[3] Pogorelić Z, Čohadžić T (2023). A bizarre cause of acute appendicitis in a pediatric patient: an ingested tooth. Children (Basel).

[4] Ismaèl LBK, Ibrahim AK, Bernadette NA (2023). Unusual cause of acute appendicitis: bone fragment. Surg Sci.

[5] Castañeda C, Valbuena D, Salamanca W, Acevedo D, Pedraza M (2022). Case report: laparoscopic management of acute appendicitis resulting from Ascaris lumbricoides. Am J Trop Med Hyg.

[6] Niang I, Dieng CK, Diouf PM (2022). "Worm within worm": acute appendicitis containing an adult Ascaris lumbricoïdes. BJR Case Rep.

[7] Wani I, Maqbool M, Amin A (2010). Appendiceal ascariasis in children. Ann Saudi Med.

[8] Agha RA, Franchi T, Sohrabi C, Mathew G, Kerwan A (2020). The SCARE 2020 guideline: updating consensus Surgical CAse REport (SCARE) guidelines. Int J Surg.

[9] Simpson J, Humes DJ (2012). Acute appendicitis. Textbook of Clinical Gastroenterology and Hepatology, Second Edition.

[10] Abukhalaf SA, Misk RA, Alzeerelhouseini HI, Irziqat IM, Asaferah AH, Aljabarein OY, Abuzaina KN (2020). Appendicitis-like picture induced by foreign body in a 2-year-old boy. Case Rep Surg.

[11] Puri R, Choudhary NS, Sud R (2013). A young female with recurrent biliary pain. J Dig Endosc.

[12] Ahmad M, Malik P, Hassan S, Dwivedi S (2015). Ascariasis presenting as hematemesis in a young boy. J Health Res Rev.

[13] Tustumi F, Hudari GG, Modolo NR, Morrell AL, de Miranda Neto AA, Dias AR (2020). Unusual cause of appendicitis. A case report of acute appendicitis caused by needle ingestion. Int J Surg Case Rep.

[14] Mohammed AA, Ghazi DY, Arif SH (2019). Ingested metallic foreign body impacted in the vermiform appendix presenting as acute appendicitis: case report. Int J Surg Case Rep.

